# Brain mechanisms involved in the perception of emotional gait: A combined magnetoencephalography and virtual reality study

**DOI:** 10.1371/journal.pone.0299103

**Published:** 2024-03-29

**Authors:** Yu-Tzu Wu, Sylvain Baillet, Anouk Lamontagne

**Affiliations:** 1 School of Physical and Occupational Therapy, McGill University, Montreal, Quebec, Canada; 2 Feil and Oberfeld Research Centre, Jewish Rehabilitation Hospital–Centre Intégré de Santé et de Services Sociaux de Laval, Centre for Interdisciplinary Research in Rehabilitation of Greater Montreal, Montreal, Quebec, Canada; 3 McConnell Brain Imaging Centre, Montreal Neurological Institute-Hospital–Montreal, Montreal, Quebec, Canada; University of California Irvine, UNITED STATES

## Abstract

Brain processes associated with emotion perception from biological motion have been largely investigated using point-light displays that are devoid of pictorial information and not representative of everyday life. In this study, we investigated the brain signals evoked when perceiving emotions arising from body movements of virtual pedestrians walking in a community environment. Magnetoencephalography was used to record brain activation in 21 healthy young adults discriminating the emotional gaits (neutral, angry, happy) of virtual male/female pedestrians. Event-related responses in the posterior superior temporal sulcus (pSTS), fusiform body area (FBA), extrastriate body area (EBA), amygdala (AMG), and lateral occipital cortex (Occ) were examined. Brain signals were characterized by an early positive peak (P1;∼200ms) and a late positive potential component (LPP) comprising of an early (400-600ms), middle (600-1000ms) and late phase (1000-1500ms). Generalized estimating equations revealed that P1 amplitude was unaffected by emotion and gender of pedestrians. LPP amplitude showed a significant emotion X phase interaction in all regions of interest, revealing i) an emotion-dependent modulation starting in pSTS and Occ, followed by AMG, FBA and EBA, and ii) generally enhanced responses for angry vs. other gait stimuli in the middle LPP phase. LPP also showed a gender X phase interaction in pSTS and Occ, as gender affected the time course of the response to emotional gait. Present findings show that brain activation within areas associated with biological motion, form, and emotion processing is modulated by emotional gait stimuli rendered by virtual simulations representative of everyday life.

## Introduction

Recognizing emotions of others is essential for effective social interactions [1]. Emotions can be conveyed through different body cues such as facial expressions, postures and body movements. The latter two would be especially useful in the context of locomotion, given that they can more easily be observed from a distance, and would thus contribute to successful pedestrian interactions [2–4]. Several neuroimaging studies, primarily with fMRI and using point-light displays (PLDs) or full-light displays, have characterized the brain regions involved in biological motion perception, i.e. the ability to perceive motion of anther individual. These studies have revealed the presence of a network comprising of the posterior superior temporal sulcus (pSTS) [5–9], human MT/V5 complex [6, 10], extrastriate body area (EBA) [9, 11–14], fusiform gyrus (FFG) [9, 12, 14–16] and temporal parietal junction (TPJ) [7, 9]. These areas would be involved in the allocation of attention and would interact with brain areas responsible for motor planning, such as the premotor cortex (PMC) and sensorimotor area (SMA) [8, 17]. Electroencephalography (EEG) studies have further provided information on the temporal course of the brain activation while exposed to PLDs, revealing the presence of two event-related responses (ERR) [18–20]. The first component (P1), occurring at ∼200ms within the extrastriate cortex, would reflect the visual onset of the moving dot pattern representing a human figure, while the second component taking place at ∼330ms in pSTS was associated with the specific analysis of motion patterns. However, the precise timing of those components appears to vary across studies due to the nature of the stimuli content or experimental paradigm, reaching latencies as long as 300ms to 600ms when performing more complex discrimination tasks, such as determining the facing direction of a PLD walker [7]. Alike the processing of facial expressions [21], the brain’s response to locomotor movement stimuli may actually span over a large temporal window following stimulus presentation, with a late ERR component involved in higher-order perception processes, such as those involved in emotional valence perception.

Using PLDs representing emotional body movements, including locomotor movements, past studies have demonstrated that emotional stimuli modulate neural activation in most areas involved in biological motion perception, such as fusiform body area (FBA) [14], the inferior frontal gyrus (IFG) [22], lateral orbitofrontal cortex (lOFC) [23], EBA [14], pSTS [14, 23–25], TPJ [23]. Among these areas, pSTS was discussed as acting as a major cortical network hub for the perceptual analysis of social cognitive cues, with reciprocal connections with the amygdala, a region involved in the processing of emotion-selective information [26, 27] that is especially responsive to threatening stimuli (e.g., negative facial expressions [14, 28, 29] or aggressive postures [30, 31]). However, no modulation of AMG activation as such has been reported in response to PLD-based emotional gait, an observation that may be explained by the lack of ecological validity of the stimuli, which could fail to elicit defensive brain activation responses in the observers [32]. To our knowledge, only a handful of studies [33–35] have investigated brain activations when observing the emotional gait of human-like agents. Alike PLD studies, a modulation of brain activation in regions such as EBA, FBA, pSTS, TPJ, IFG was observed, but additional effects in the AMG and the insula were also reported, suggesting that the display of a virtual human-like agent recruits a broader set of cortical and subcortical areas compared to PLDs. The perception of the expressed emotion was also reported to be more accurate when an actor/human-like agent with physical traits is presented compared to PLDs [2], which could affect underlying brain activation. Furthermore, the modulatory effects of different emotional valences of gait on brain activation has been seldom studied given the merge of negative and positive valence of stimuli in the published studies [33, 35]. A single study conducted by Schneider et al. (2014) that used functional near-infrared spectroscopy examined the effect of valence and pointed out a selective increase of AMG activity only in response to negative (e.g., sad, fearful, or angry) and not positive (e.g. happy) emotion gait stimuli portrayed by human-like agents [34]. Altogether, further evidence is needed to fill a knowledge gap on the modulatory effect of emotional valence of gait patterns on brain activation, and information on the temporal characteristics of such modulation is crucially lacking. In addition, studies that displayed human-like agents all used the same set of animations created by Roether et al. (2009) [36], which feature a uniform, gender-neutral grey mannequin body overlaid on motion capture data of male and female actors. Thus, these animations not only have limited realism, but they may also bear a confounding effect of gender on emotion perception. Indeed, growing evidence shows that the information about gender and emotion conveyed by biological motion stimuli interact and can modulate the observer’s perception. Supporting this idea, Johnson et al. (2011) observed that angry throwing motions depicted via PLDs were overwhelmingly judged as performed by men [37]. Zibrek et al. (2015) also observed that angry gait was most often rated as “male”, regardless of whether the motions were that of a male or a female agent [38]. At the neurological level, Kret et al. (2011) showed that male vs. female threatening facial expressions elicited differential activations within the EBA, FBA, pSTS, and PMC, those areas being more active while exposed to male vs. female-threatening stimuli [39], which suggests that gender modulates the brain response to emotional stimuli. To our knowledge, whether gender and emotional gait stimuli may act in interaction to modulate brain activation remains to be elucidated. Advancing knowledge about such interaction may provide novel insights into pedestrian interactions in community settings. Finally, whether we consider PLD or human-like agent studies, there exists an inadequate knowledge of temporal characteristics of brain signals in response to emotion gait stimuli and dynamic body expressions as a whole.

Here we designed three-dimensional (3D) human-like female and male virtual agents that expressed positive and negative emotional states through their locomotion. We sought to examine how the emotion and gender of the virtual agents affected time-resolved brain activations in a group of male and female observers using magnetoencephalography (MEG) source imaging. Our primary objectives were to test for differential activations in brain regions involved in the processing of different emotional valences displayed by the locomotor movements of virtual pedestrians and to characterize their neural dynamics via ERRs. Our secondary objective was to determine whether the gender of the virtual pedestrians affected those brain responses. We hypothesized that: i) gait emotional valence affects late ERR components (>300ms) while leaving earlier ERR components (e.g., P1) unchanged; ii) the activation of the amygdala, EBA, FBA, pSTS, and lateral Occ is enhanced by emotional gait stimuli, with negative emotion (e.g., anger) evoking larger brain responses compared to positive emotion (e.g., happiness); iii) male pedestrians evoke enhanced brain activations compared to female pedestrians, especially when moving with an angry gait pattern, which is typically perceived as more threatening [40–42].

## Methods

### Participants

Twenty-one right-handed healthy young adults (10 males and 11 females; Asian: 8, Caucasian:6, Indian: 2, Arabic:2 and other: 3) aged 18–29 years (24.82 ± 2.90 years [mean ± 1SD]) participated in this study. They were recruited between July 19^th^, 2021, and December 22^nd^, 2021. Individuals with a current or past neurological or psychiatric disorder were excluded, as well as those with an implanted device interfering with MEG acquisition. The study was approved by the McGill University Health Centre Research Ethic Board, and all participants gave their written informed consent prior to entering the study. Authors had access to information that could identify individual participants during and after data collection.

### Stimulus presentation

**Fig 1:**
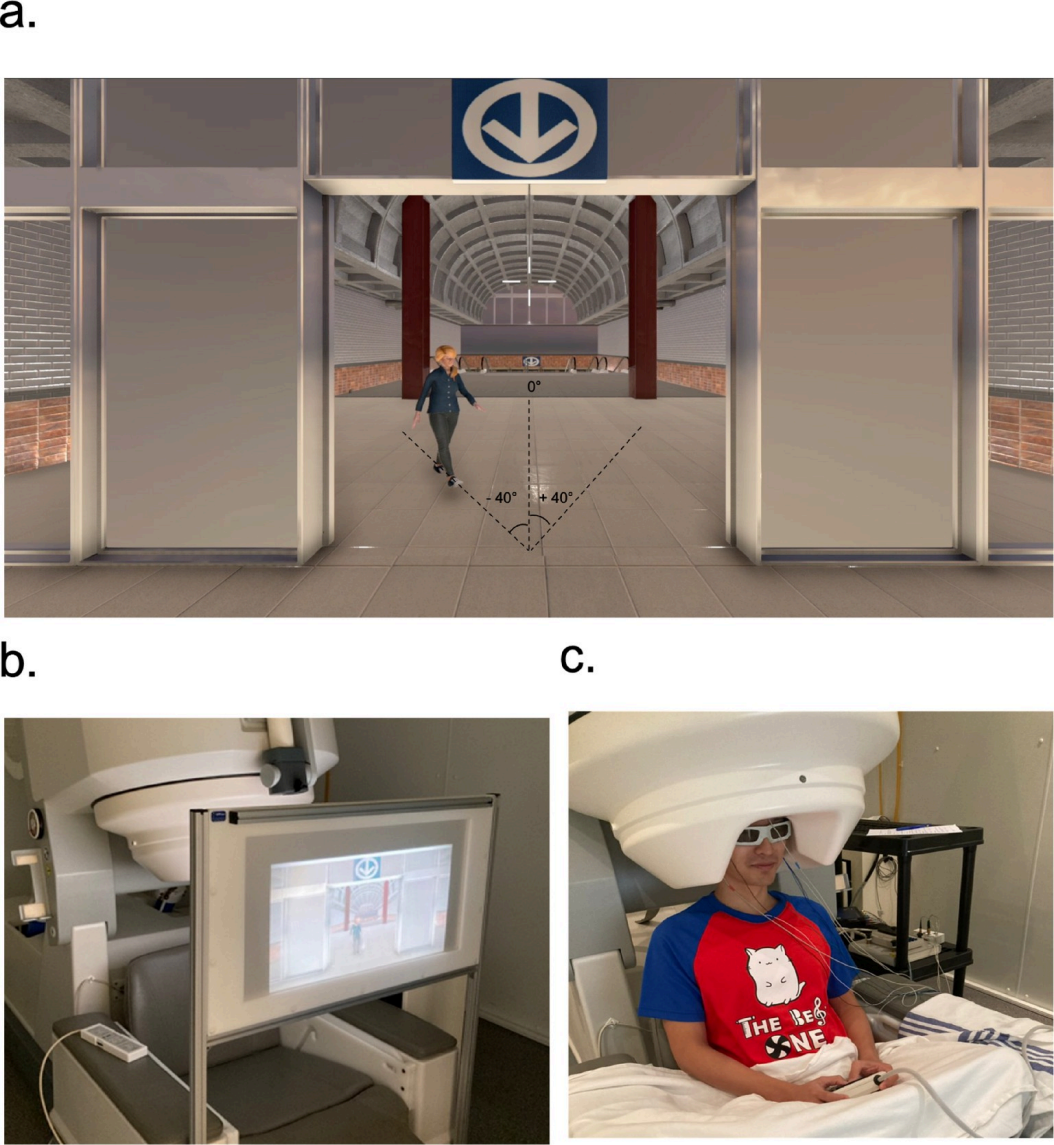
(a) Example of the virtual environment with a metro station background and an approaching virtual pedestrian walking with an emotional gait. The black dotted lines (not visible to the participant) indicate the three possible pedestrian trajectories. (b) The experimental setup, including the rear-projection display and the MEG instrument. (c) One participant with electrodermal sensors measuring heartbeats and eye movements, as well as 3D glasses. They were instructed to hold the response box with both hands to avoid MEG signal contamination induced by muscle tension. Reprinted from [47], under a CC BY license, with permission from ProQuest Dissertations & Theses (McGill University), original copyright Yu-Tzu Wu 2022.

The stimuli consisted of short movie clips displaying three emotional gait patterns (angry, happy, neutral) performed by a female and a male virtual pedestrian (Fig 1A). These emotional states were chosen given their negative (angry), positive (happy) and neutral valences, and as they show relatively similar walking speeds (as opposed to a sad gait, for instance, which would be slower [43, 44]), hence preventing a potential confounding effect of ‘speed of motion’ when contrasting brain activation across emotional gait conditions. They were shown walking toward the observer’s vantage point, from either the top middle (0°), right, or left (±40°), in a Montreal subway station, and disappeared when reaching the midline of the door frame. A centrally located crosshair (0°) was overlaid for participants to fixate on during each trial. Emotional gait animations were captured from one male and one female actor portraying whole-body expressions of the different emotions of interest while walking on a self-paced treadmill (walking velocities ∼1.6 m/s). Their full body kinematics were recorded at 120Hz using a Vicon™ motion capture system and 40 passive reflective markers positioned as per the Plug-In-Gait model from Vicon™. Pegasus Advanced™ was used to animate a male and a female virtual pedestrian with the recorded movements. Male and female virtual agents with a static neutral facial expression were selected and a blurring effect was further applied over the facial area to avoid attention to be drawn towards the virtual pedestrian’s faces [38]; therefore, no facial expression could be detected. A total of 4 video clips each displaying a gait animation comprising of four gait cycles were extracted per actor/actress (2 men and 2 women) for each emotional gait condition. These trials were validated a priori on a sample of 22 healthy participants (female = 9, male = 13, age = 27.25±4.36 years [mean±1SD]) that were different from those participating in the present study. The validation process involved 72 emotional gait trials (12 per emotion and gender) that participants viewed on a computer screen, and for which they identified the emotions according to the following response options: happy, neutral angry. Amongst the animations that yielded an accuracy of ≥ 80%, the two with the highest accuracy for each emotion were selected for this MEG experiment. Having two rather than single animations enabled variability from trial to trial, akin to real-life experiences, while minimizing the risk of identifying the emotion based on other unrelated information. The 3D virtual environment (VE), programmed in Unity 3D™, was projected using a PROPixx™ (VPixx Technologies) projector on a rear-projection screen (50cm height x 80cm length) located in the MEG room (Fig 1B). The VE animations were displayed with a resolution of 1920x1080 pixels and a refresh rate of 60 Hz.

### Task

**Fig 2:**
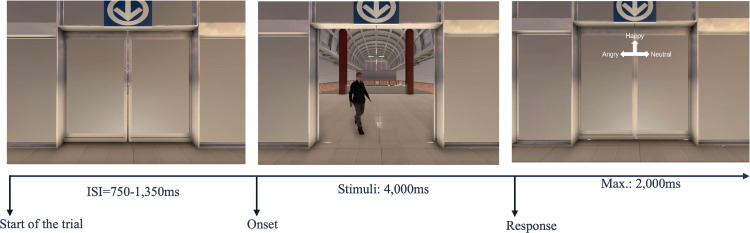
Timeline of an experimental trial with an example of a male pedestrian approaching from the left. Initially, the sliding door was closed, and the pedestrian was not visible to the participant (left panel). Then, the door opened and the observed would see an approaching pedestrian with an emotional gait pattern (middle panel). At the end of the trial (right panel), a display with three response options was overlaid to the virtual environment to request participants to provide responses. Abbreviation: ISI, inter-stimulus interval. Reprinted from [47], under a CC BY license, with permission from ProQuest Dissertations & Theses (McGill University), original copyright Yu-Tzu Wu 2022.

The entire experiment lasted approximately 2.5 hours, including preparation and data collection, and the MEG scanning itself lasted about one hour. First, participants were requested to fill out the Positive and Negative Affect Schedule (PANAS) questionnaire to evaluate their mental state [45]. Subsequently, a minimum of twenty practice trials were conducted before MEG scanning until participants reached a minimum of 80% emotion discrimination accuracy and felt comfortable with the task. Participants were then assessed while seated under the MEG helmet, holding a small response box consisting of keys providing their responses to the task. The response box was held with both hands resting on their lap to reduce MEG signal contamination induced by arm muscle tension (Fig 1C). As shown in Fig 2, each stimulus depicting a virtual walking pedestrian lasted 4000ms, followed by a response prompt screen. If no response was provided by the participant within 2000ms, a new trial was initiated. Trials were separated by an inter-stimulus interval of variable duration between 750 and 1350ms. A total of 480 trials were collected, with 80 trials for each of the six combinations (3 emotions x 2 gender). Within each of the 10 blocks of 48 trials, the presentation order of emotions and directions of approach was also randomized. Participants were instructed to always fixate a crosshair at the center of the display and to report the response when prompted by the response screen with a 3-alternative response choice, identified with text (happy, neutral, angry) and arrows pointing up, left, or right. The position of response options on the response screen was fixed within a given block but was randomly arranged between blocks to maintain participant’s alertness. It also potentially dissociates brain activation involved in the emotional processing from that involved in the preparation of the directional button presses. Each block lasted 5mins, and participants were invited to rest for 2mins every two blocks. Upon completing MEG data collection, the participant responded to a short feedback questionnaire (SFQ) about their perception of the VE [46].

### MEG data acquisition

MEG data were acquired at the McConnell Brain Imaging Centre, Montreal, Canada, using a 275-channel whole-head system (CTF/VMS, Port Coquitlam, British Columbia, Canada). Data were recorded with a sampling frequency of 2400Hz. Prior to MEG data collection, each participant’s scalp, eyebrows, and nose were digitized with approximately 200 points using a 3D digitizer system (Isotracker, Polhemus) to co-register functional MEG data with the default anatomy (MNI ICBM152 [48]) provided in Brainstorm [49]. The participant’s head position inside the MEG sensor helmet was determined with three localization coils fixated at the nasion and left/right preauricular sites (fiducial points). Horizontal and vertical electrooculograms and an electrocardiogram were acquired with bipolar montages to capture eye movements, blinks and heartbeats, respectively, for subsequent MEG artifact detection and removal.

## Data analysis

### Behavioral data

Emotion discrimination accuracy score (n = 21) was quantified in terms of the proportion of correct responses (%), regardless of the approaching direction of the pedestrians, and were analyzed with a generalized estimating equation (GEE) model comprising two within-subject factors: emotion (angry, happy, and neutral) and gender (female and male). In addition, and in order to detect the presence of a possible learning effect on response accuracy, a linear mixed model was conducted across the 10 consecutive blocks of trials using participants’ response during the experiment. Statistics of post-hoc comparisons were corrected using Bonferroni correction. The SFQ and PANAS scores were summarized with descriptive statistics as mean ± 1standard deviation (SD).

### MEG data preprocessing

MEG data preprocessing and analysis were conducted using Brainstorm application [49]. The data were resampled at 256Hz, and notch filtered at the power frequency of 60 Hz with its first three harmonics to reduce power line contamination. The power spectrum density (Welch’s method) of all sensor traces over the entire recordings was estimated to evaluate noise levels. A bandpass filter was subsequently applied at 0.1-30Hz to remove high-frequency components. Artifacts related to eye movements and cardiac activity were automatically detected and attenuated using signal space projections. Independent components analysis of the sensor time series was performed, resulting in 20 components obtained with the extended infomax algorithm. Topographical maps of possible artifactual components were visualized and manually eliminated. MEG data were then segmented into epochs ranging from −500 to 4000ms and the baseline correction was referenced from -500 to 0ms (stimulus onset: door opening). Only correct trials in terms of emotional discrimination were considered for further analysis.

### MEG source estimation

Source maps were obtained from the event-related trial average data for each participant and condition. Head models were obtained using the overlapping-sphere approach. The MEG source maps were constrained to 15,000 vertices distributed over the cortical surface. Prior to each MEG session, instrumental and environmental noise in the empty MEG room were captured with a 2-min recording and summarized via a noise covariance matrix subsequently used in the source mapping procedure. We used Brainstorm’s dynamic statistical parametric mapping (dSPM) estimator of cortical current density with default parameters (depth weighing order set to 0.5 and maximal amount 10) [50]. The dSPM source maps are standardized by the equivalent of the MEG signal-to-noise ratio at each cortical location. Spatial smoothing of the resulting source maps in each participant and each task condition was applied, with a full width at half maximum parameter of 3 mm. Five bilateral regions of interest (ROIs), amygdala (AMG), fusiform gyrus (FBA), extrastriate body area (EBA), lateral occipital cortex (Occ), and the posterior superior temporal sulcus (pSTS), were selected according to previous studies of biological motion perception and emotion processing [5, 6, 33]. The Montreal Neurological Institute coordinates [x, y, z] of each ROI location were AMG (L[-32.9, -18.5, -12.5]/R[33, -21.7, -15.2]), FBA (L[-38.4, -26.6, -30.9]/R[35.5, -36.2, -27.9]), EBA (L[-53.5, -74.4, 1.3]/ R[58.7, -66.2, 8.8]), pSTS (L[-66, -52.8. 21.1]/R [70.7, -30.1, 10.3]), and lateral Occ (L[-39.4, -82.0, 7.9]/R[37.5, -91.6, 6.4]). The surface area of each ROI comprised between 60 and 120 vertices, corresponding to 7–16 cm^2^. For each condition, a time series estimated currently at each cortical source within each ROI was extracted and used for the subsequent ERR analysis. We also derived cortical source contrast maps in angry vs. neutral and happy vs. neutral by computing the difference between rectified cortical maps for each subject before averaging across participants.

### ERRs analysis

We computed the mean response from each ROI per hemisphere from the dSPM maps. A z-score transformation was applied to the resulting ROI traces with respect to their respective pre-stimulus mean and standard deviation. We considered two ERR components of interest, P1 and LPP, and estimated the latency of their respective peaks from the grand average ROI waveforms across participants and conditions. The P1 component was defined as the maximum positive peak after stimulus onset, with ROI-specific latencies: AMG, FBA, and EBA: 200-250ms, lateral Occ: 250-300ms, and pSTS: 250-350ms. For LPP, we used a time interval between 400ms and 1500ms after stimulus onset, in line with previous studies [51–54]. Furthermore, and since the brain responses involved in emotion processing within LPP varies over time, dividing the LPP phase into smaller time windows was recommended [53, 54]. More specifically, and based on Stolz et al. (2018) which involved immersive virtual reality and the analysis of brain activity in response to virtual avatars with facial emotions [53], LPP in the present study was split into an early (400-600ms), a middle (600-1000ms), and a late LPP component (1000-1500ms). Such time windows enhance the comparability with previous studies [52–54] while accounting for the time course of the observed brain responses in our experiment. The LPP in the present study was quantified around its peak amplitude as well as during sustained activity for all three phases. The mean amplitude of source signals was derived across each LPP time window.

### Statistical analysis

Statistical analyses for MEG and behavioral data were conducted by means of generalized estimating equations (GEE) in SAS 9.4, unless otherwise specified below. We first verified with univariate analyses that there were no hemispheric differences in P1 or LPP. We, therefore, pooled source data from the homologous ROIs in both hemispheres for all subsequent analyses. P1 was analyzed separately for each ROI with a GEE model comprising of emotion and gender as within-subject factors. For LPP, a GEE model with three within-subject factors, i.e., emotion, gender, and phase (early, middle, and late), was used. Effects were deemed statistically significant at p<0.05, corrected for multiple comparisons with Bonferroni corrections. Statistical differences in cortical contrast maps were assessed using two-sample paired parametric t-tests with Brainstorm, with a p<0.05 significance level and a false discovery rate correction. Response accuracy on the emotion identification task was conducted by means of a GEE model with two within-subject factors (emotion and gender). In addition, and in order to detect the presence of a possible learning effect on response accuracy, a linear mixed model was conducted across the 10 consecutive blocks of trials using participants’ response during the experiment. In the absence of established approaches to calculate sample sizes for GEEs, the sample size calculation for this study was based on a repeated-measure analysis of variance with two within-subject factors (emotions, gender) with P1 as main outcome. The estimation was conducted in G*Power 3.1, while considering a small-to-medium effect size of 0.25, a power of 80% and level of significance of 0.05, which yielded a sample size of 19. To account for possible dropouts or technical issues, a sample size of 21 participants was considered.

## Results

### Behavioral data analysis

**Fig 3:**
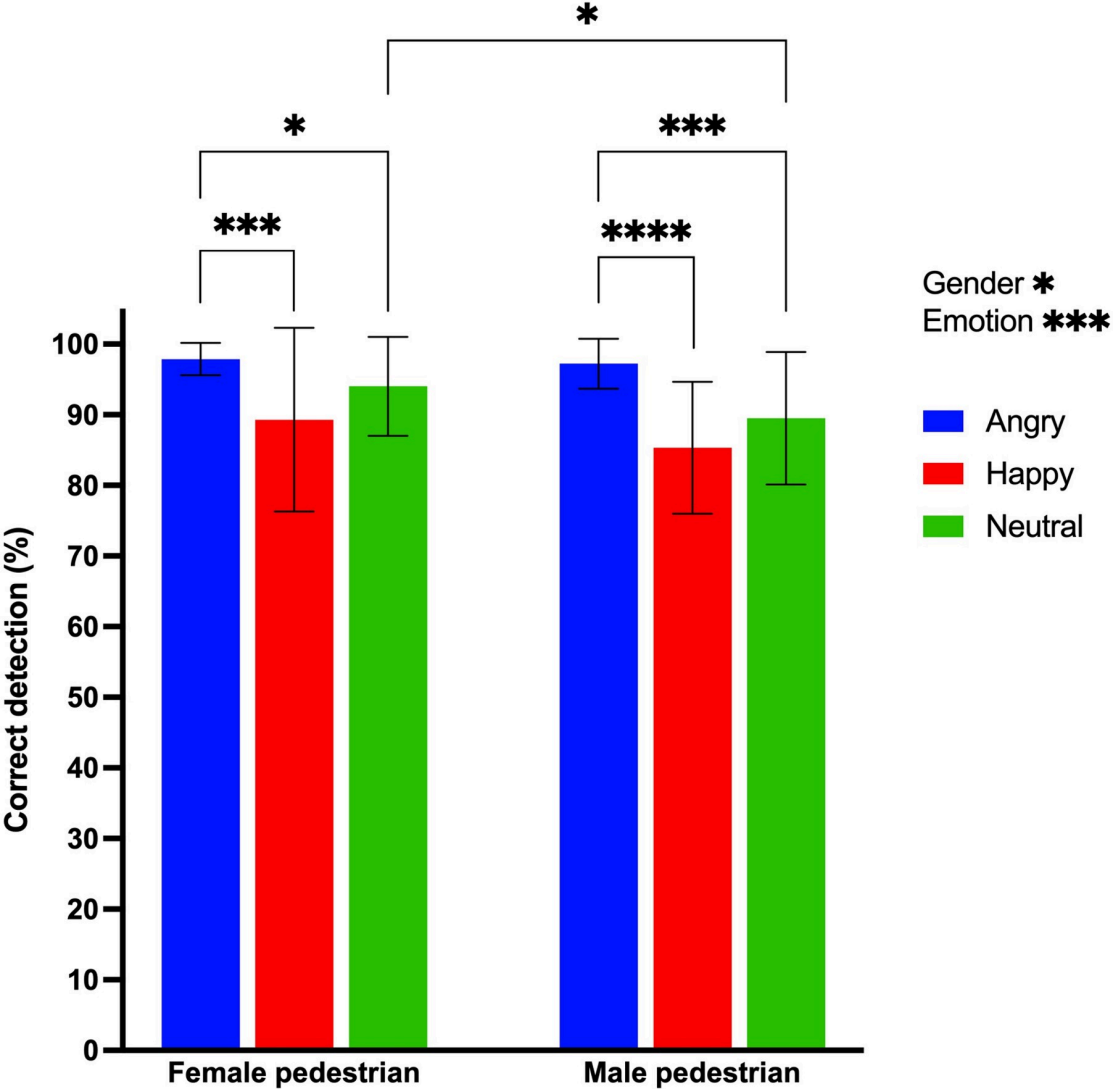
Mean proportion of correct responses (± 1SD) in the emotion identification task for each emotional gait condition (blue: Angry; red: Happy; green: Neutral) for the female (left column) and male virtual pedestrian (right column). Statistically significant main and interaction effects are indicated, as applicable. Likewise, post-hoc comparisons that were statically significant are also illustrated (* p<0.05. ** p<0.01. *** p<0.001. **** p<0.0001).

As shown in Fig 3, participants correctly identified the emotions associated with gait with at least 80% accuracy across conditions. Significant main effects of emotion (χ2(2, 126) = 16.38, p<0.001) and gender (χ2(2, 126) = 4.26, p = 0.039) were observed. Post-hoc comparisons further revealed significantly higher accuracy of response for the angry vs. neutral and happy gait (p<10^−3^), as well as for the female vs. male pedestrian (p = 0.021). There were no significant differences in response accuracy across blocks during the experiment (p = 0.812). No participant showed a strong positive (31.1±7.1) or negative affect (17.0±5.3) on the day of testing on the PANAS (max = 50). The SFQ score was 3.68 ± 0.61 out of 5, indicating a moderate sense (range: 3.0–4.0) of presence in the VE.

### MEG source analysis

**Fig 4:**
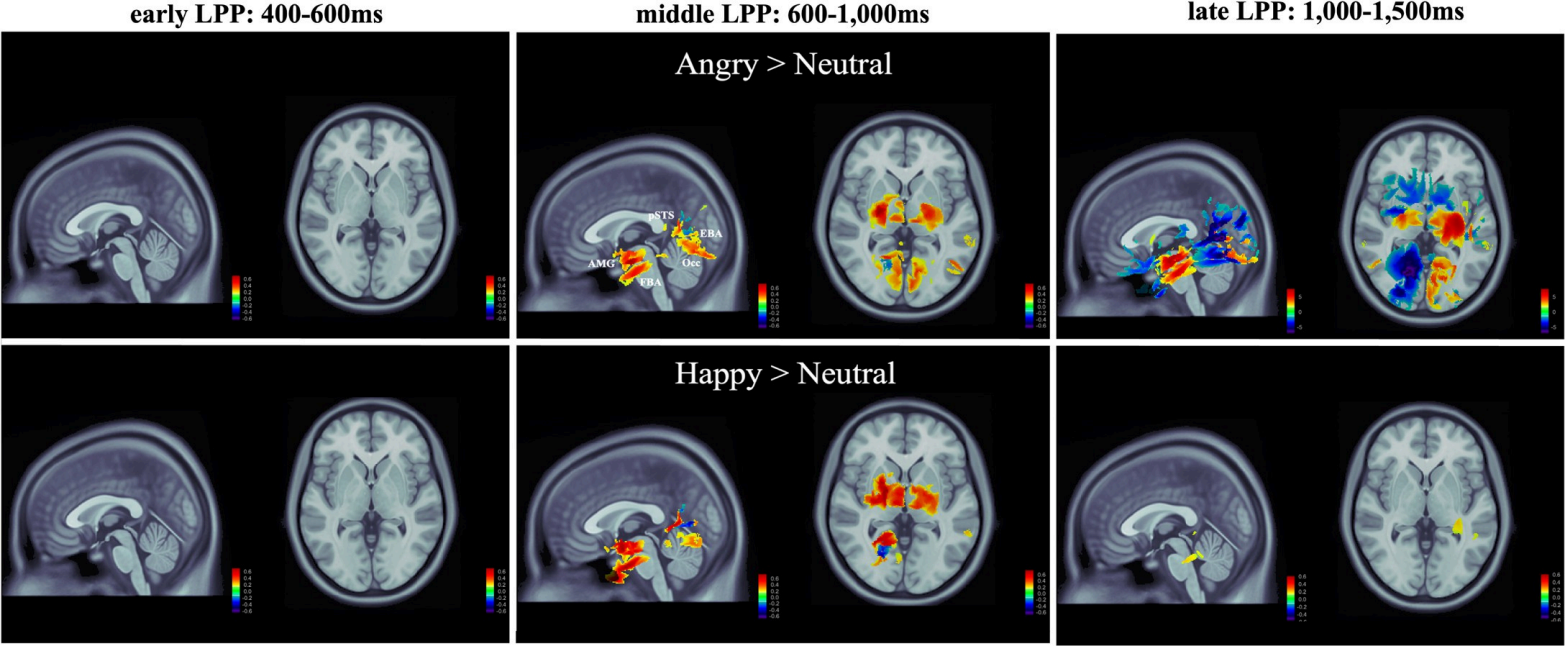
Contrasts of emotional (upper: Angry vs neutral; bottom: Happy vs neutral) gait patterns showed stronger and more widespread emotion-specific activation (left: Medial view; right: Superior view), and the ROIs were indicated in the activation areas. The colour bars on the right indicate the grading of the z-score. Note that the scale of angry vs. neutral (top-right) was modified to match the values. The obtained results were averaged across time interval corresponding to early LPP (400-600ms), middle LPP (600–1,000ms), and late LPP (1,000–1,500ms), respectively. Abbreviation: pSTS, posterior superior temporal sulcus; FBA, fusiform body area; EBA, extrastriate body area; AMG, amygdala; Occ, lateral occipital cortex.

**Fig 5:**
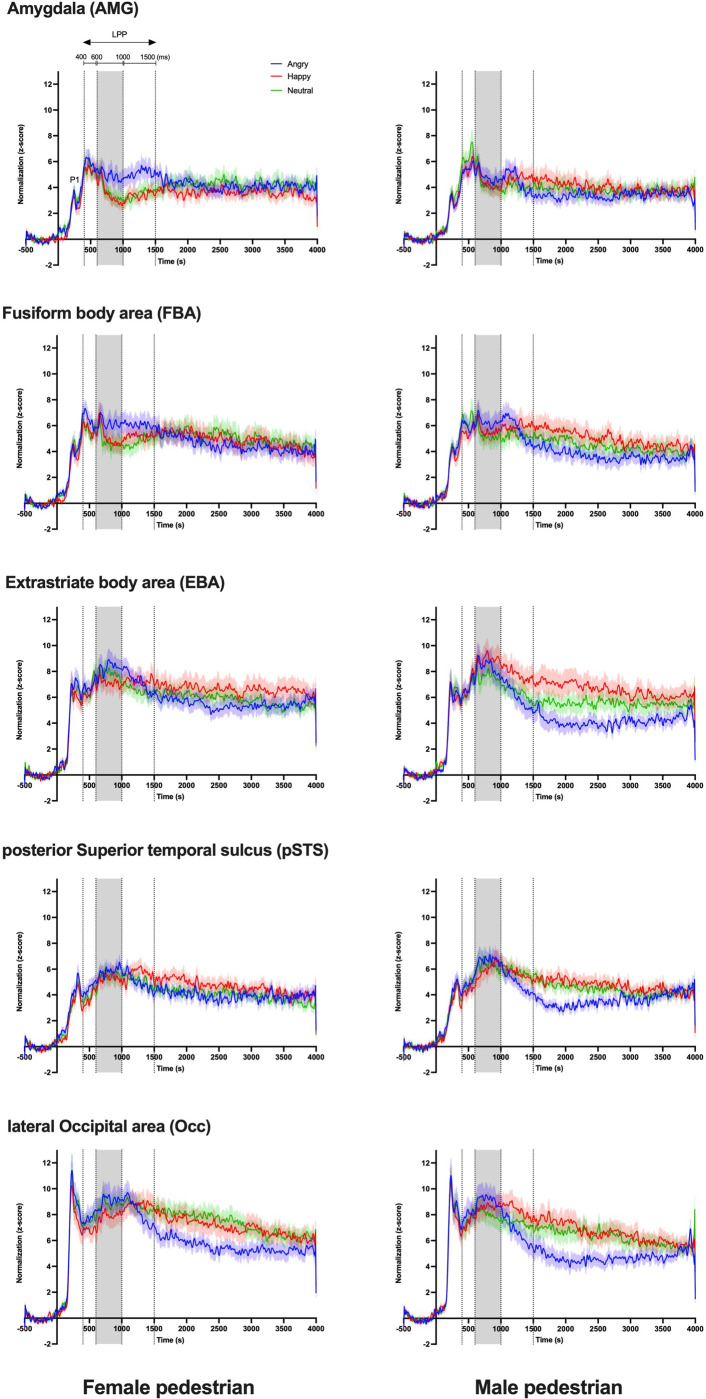
Each trace represents the grand-average waveform with 1 SEM for each emotional gait condition of the female and male pedestrians. The different colours illustrate the different emotions, i.e., angry (blue), happy (red), and neutral (green). The three separate time intervals of the entire LPP component are marked with vertical lines representing the early (400-600ms), middle (600-1000ms), and late LPP (1000-1500ms) phases. Abbreviation: SEM, standard error of the mean.

**Fig 6:**
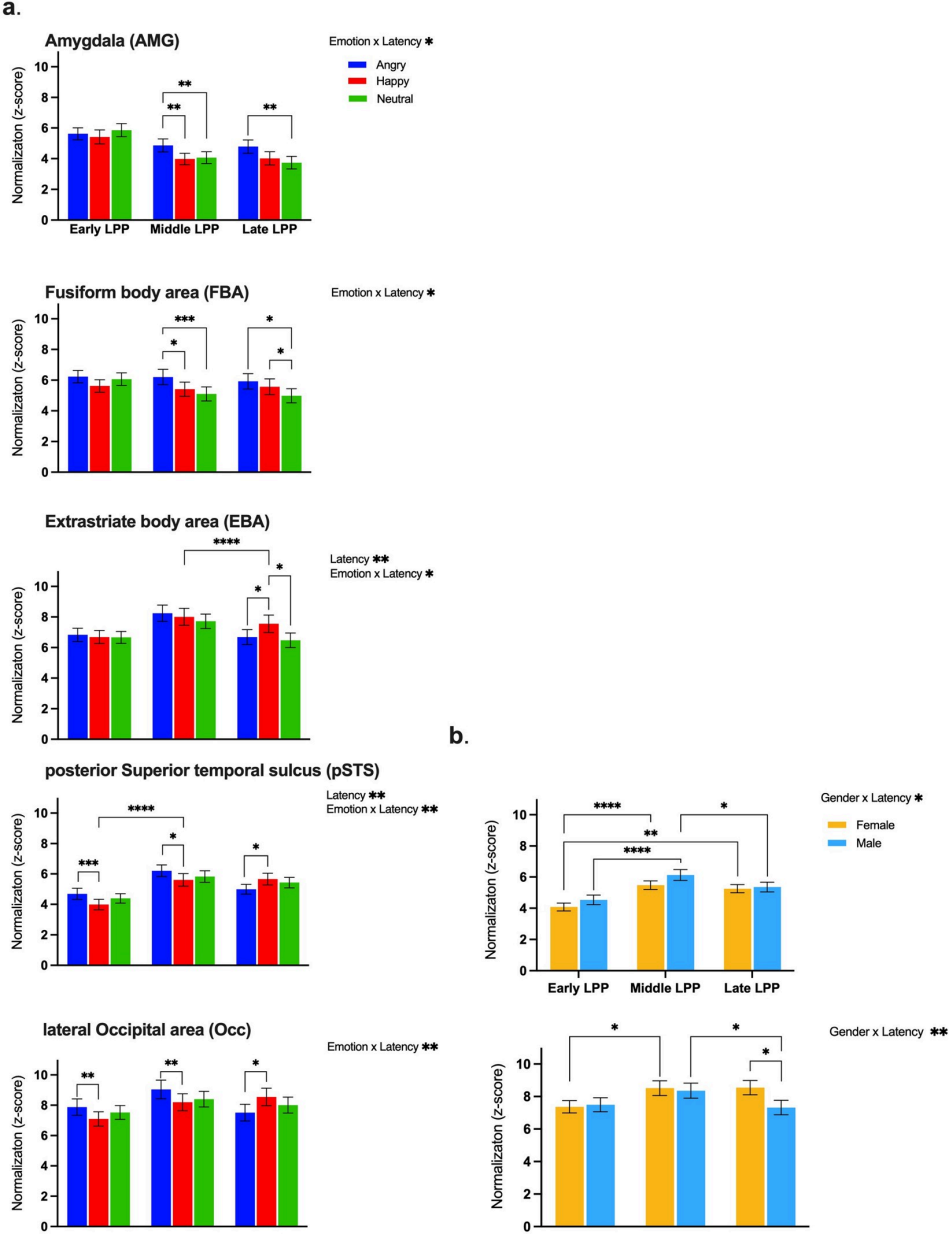
(a) Mean LPP amplitude ± 1 SEM for three separate time windows over 5 ROIs with significant emotion x phase interactions. (blue: angry; red: happy; green: neutral). (b) Mean LPP amplitude of pSTS and Occ with the significant gender x phase interactions (orange: female pedestrian; light blue: male pedestrian). The symbol indicates * p<0.05. ** p<0.01. *** p<0.001. **** p<0.0001. Abbreviation: pSTS, posterior superior temporal sulcus; FBA, fusiform body area; EBA, extrastriate body area; AMG, amygdala; Occ, lateral occipital cortex; SEM, standard error of the mean.

The activity in ROIs was first evaluated using t-tests in Brainstorm, which allowed identifying significant levels of activation in response to emotional gait conditions with respect to the neutral condition. As illustrated in the contrast maps in Fig 4, key regions involved in emotion processing and/or biological motion perception (e.g., AMG, FBA, EBA, pSTS, and Occ) showed significantly enhanced activation and confirmed the selection of ROIs for the present experiment. Furthermore, ERR traces, as shown in Fig 5, retrieved from the five selected ROIs showed two evoked components, P1 and LPP, at latencies of ∼200ms and ∼400ms, respectively. GEE analyses revealed that the amplitude of P1 remained unaffected by emotion (χ2(2, 252)<2.38, p>0.305) and gender (χ2 (1,252)<0.44, p>0.508) and showed no statistically significant emotion x gender interactions (χ2 (2,252)<1.90, p>0.387) across the five ROIs. As observed in Fig 6, however, the mean LPP amplitude for the five ROIs showed substantial variations across emotional gait conditions and time intervals (summarized in Table 1).

**Table 1:** Main and interaction effects on LPP amplitudes in each ROI. Statistically significant results are indicated with star symbols, when applicable: *p<0.05, **p<0.01, ***p<0.001. Abbreviations: pSTS, posterior superior temporal sulcus; FBA, fusiform body area; EBA, extrastriate body area; AMG, amygdala; Occ, lateral occipital cortex; df, degree of freedom; Pr, probability.

	*Main effect*									*Two-factor interaction*								
	df, Chi-square, Pr. > ChiSq.																	
	Emotion			Gender			Phase			Emotion x Phase			Emotion x Gender			Phase x Gender		
***AMG***	2	3.94	0.1392	1	0.76	0.3822	2	4.99	0.0825	4	10.39	0.0344^*^	2	3.02	0.2205	2	0.60	0.7399
***FBA***	2	5.77	0.0559	1	0.01	0.9254	2	0.67	0.7162	4	12.00	0.0173^*^	2	1.51	0.4695	2	1.88	0.3911
***EBA***	2	1.30	0.5219	1	0.82	0.3638	2	10.51	0.0052^**^	4	10.94	0.0272^*^	2	2.92	0.2326	2	4.99	0.0823
***pSTS***	2	1.19	0.5511	1	1.52	0.2170	2	11.90	0.0026^**^	4	14.16	0.0068^**^	2	1.61	0.4475	2	7.06	0.0293^*^

The first key finding is that, while there was no significant main effect of the emotional gait on LPP amplitude in any of the five ROIs, the LPP amplitudes showed a significant emotion x phase interaction in all those ROIs. Post-hoc comparisons, further detailed in Fig 5, revealed that in AMG and FBA, angry pedestrians evoked significantly larger LPP responses in comparison to the happy and/or neutral pedestrians in the middle (p<0.001–0.0091) and late LPP phases (p = 0.0031–0.0120), but not in the early LPP phase (p = 0.0749–0.6850). In EBA, a larger response was elicited by the happy pedestrian in comparison to both the neutral and angry pedestrians in the late LPP phase (p = 0.0139–0.0324). Overall, larger responses during the middle phase of EBA were observed compared to its later phase. In pSTS and Occ, the angry pedestrian elicited significantly larger LPP responses compared to the happy pedestrian but not the neutral pedestrian (p = 0.0521–0.2956) in both the early (p<0.001–0.0065) and middle LPP phases (p = 0.0092–0.0214). In the late LPP phase, a significantly weaker response for the angry vs. happy pedestrian was instead observed due to a dramatic decrease in signal amplitude (p = 0.0341–0.0498).

For clarity, the effects of gender, while considered in the same GEE model as emotion and phase, are presented separately in Fig 6B. No significant main effect of gender only or gender x emotion interactions on LPP amplitude was observed in any of the ROIs (p = 0.0823–0.0740). Significant gender x phase interactions, however, emerged for pSTS and Occ specifically (p = 0.0033–0.0293). In those two ROIs, the interactions were caused by subtle variations in the way the signal amplitude varied across LPP phases for the male vs. female pedestrian conditions. Indeed, while either or both the male and female pedestrians elicited larger signal responses in the middle vs. early LPP phase (p<0.001–0.0453), only the male pedestrian condition showed larger signal amplitudes in the middle vs. late LPP phase (p = 0.0101–0.0190). In Occ, however, a larger response was also evoked in the late LPP by the female pedestrian compared to the male pedestrian (p = 0.0104).

## Discussion

MEG was used to investigate the spatiotemporal dynamics of brain activity when healthy young observers are exposed to emotional gait stimuli portrayed by virtual male and female pedestrians in a virtual community environment. Our primary objective was to characterize the brain signal changes involved in emotion perception from locomotor movements. As hypothesized, the richly textured ecological simulations used yielded a modulation of brain activation in response to emotional gait in ROIs related to visual processing (e.g., lateral Occ.), motion and form analysis (e.g., pSTS, EBA, and FBA) and processing of social cues (e.g., AMG). This pattern of modulation is overall consistent with earlier studies that used simplified/impoverished stimuli, where effects of the emotional movement were also detected in brain areas related to body representation (i.e., EBA, FBA, and pSTS) [14, 24, 55]. Such modulation further aligns with the involvement of those brain structures in the action-observation network (e.g., pSTS and lateral OCC) [56]. In addition, it takes place within brain structures that have been associated with the “theory of mind” (e.g., pSTS, AMG, and Occ), a mentalizing process critical for social interactions that allows to understand or infer the mental states of others [57].Together, these observations support the idea that the modulation of brain activation observed in the present study serves the purpose of inferring and interpreting affective states others’ locomotor movements. A noteworthy difference with previous PLD studies, however, is that we observed an emotion-dependent activation in the AMG. For example, enhanced AMG activation was previously observed in response to biological vs. random motion (e.g., object or scramble motion) [58, 59]. However, we are not aware of modulations of AMG activation in response to emotional gait stimuli induced by PLD stimuli, which might be explained by the extent of ecological validity of the stimulus employed [32]. Here, larger AMG activations were elicited by the virtual pedestrian displaying an angry gait. Similarly, a previous fMRI study conducted by Goldberg et al. (2015) also observed AMG activations in response to negative emotional gait stimuli displayed by faceless mannequins, which, while not as realistic as those employed in the present study, had enhanced ecological validity compared to PLDs [33].

We also observed modulations of FBA and EBA activation in response to a happy gait. We are currently not aware of PLD or full-light display (FLD) studies showing the influence of a happy gait on brain activation. A fNIRs study [34] with the presentation of faceless virtual agent showed no significant differences in brain activation in areas EBA, FBA, pSTS, and TPJ between happy vs. neutral gait conditions, and their reported correct responses in the happy (74%) and neutral (60%) gait stimulus conditions were lower than those from our present data (87.3% and 91.7%, respectively). We interpret this difference as indicative of potential ambiguity in emotion recognition on behalf of participants, which might have reduced differential effects in brain activations between the two emotional gait conditions in previous studies. We noted that two studies on emotional body movements in other tasks, however, did report enhanced FBA and EBA activations in response to happy stimuli, as in our present study. The tone that used PLDs reported high emotion recognition rates (above 80%) [14], and the that used FLDs reported significant differences in subjective rating between emotional and neutral gaits [55]. This suggests that the salience of the stimulus used is a crucial component of the paradigm. The human-like virtual agents with physical appearances and displacements in space embedded in a realistic environment enabled participants to reminisce their own real-life experiences, which might influence brain responses to emotionally charged gait movements.

Two main ERR components, P1 (∼200ms) and LPP (∼400ms-1500ms), were observed, and we verified our hypothesis that the presence of an emotional (non-neutral) gait stimulus did not modulate the P1 amplitude. Previous efforts did not report changes in P1 amplitude in response to PLD walkers vs. scrambled motion [60, 61]. This absence of effect was discussed as the P1 component being related to early visual (e.g., luminance) or lower-order motion processes (e.g., basic structure cues or selective spatial attention) [62]. We did find a second component (LPP) that is considerably delayed compared to what is reported in the literature (∼300ms) on biological motion perception [60, 61]. A previous MEG study examined brain responses in an identification task of the facing direction of PLD walkers and reported a second component between 300ms and 600ms post-stimulus onset [7]. We argue that the latency of this second component may depend on task complexity. In fact, the time window of our second component ranged from 400 to 1500ms, is consistent with that of LPP, which is typically associated with selective attention to the emotional contents of stimuli [63].

We found an interaction between emotion and the phases of this LPP component across ROIs, which suggests that the dynamics of the LPP are modulated by the emotion presented. Previous studies have shown that the emotional content (e.g., affective pictures [64–66], faces [67, 68], and hand gestures [69]) robustly potentiates the LPP. Our results indicated that in the early LPP phase, pSTS and Occ elicited larger responses to angry vs. happy gaits. We note that the pSTS has been linked preferentially to motion and social processing [8, 16, 70–73]. Schneider et al. (2014) also found that the STS showed enhanced activation in response to negative vs. neutral stimulations [34]. During the middle LPP phase, the effect of angry gait in Occ and pSTS persisted, and we found a subsequent enhancement of brain activation within AMG, FBA, but not EBA, for the angry vs. happy and/or neutral gait. We, therefore, conclude that the processing of bodily-conveyed emotions is organized in a hierarchical manner, as described by Sokolov et al. (2018) [74]. Visual information is processed in pSTS and Occ, then transferred to the AMG, FBA, and EBA for subsequent analysis of emotional contents [24, 75, 76] via known anatomical connections [77–79]. We did not find an effect of selectivity for the angry gait condition in the middle LPP phase in EBA. We speculate that this region may be dominantly involved in the processing of motion over emotion [13]. In the late phase of the LPP, we found an enhanced neural response for angry vs. neutral gaits exclusively in the AMG. Such enhanced and persistent AMG activation in response to negative stimuli is consistent with earlier work showing that AMG is principally involved in the processing of stimuli related to threats and danger [80–83]. Interestingly, the happy gait condition also induced larger activations in the late stage of LPP in FBA and EBA, compared to neutral gait stimuli. The reasons for this late enhancement of LPP in the happy condition are still unclear. This late LPP effect may have been caused by the recognition uncertainty for happy vs. neutral stimuli [33, 84, 85]. Such uncertainty would imply longer decision-making processing time and hence delayed responses. It may therefore explain the prolonged late LPP phase for the happy condition, as opposed to the angry condition since this latter might have been easier to detect.

Our secondary objective was to determine whether the gender of virtual pedestrians affected the brain’s response to emotional gait stimuli. We expected larger brain activity in response to male vs. female pedestrians, with the angry male condition eliciting larger activations in all task conditions. Our results, however, revealed no differences in the emotion-dependent modulation of LPP amplitude when viewing male vs. female pedestrians in any of the tested ROIs. This observation differs from an fMRI study conducted by Kret et al. (2011), which found significantly higher activation in EBA and STS when viewing threatening male vs female actions [39]. In that study, however, the angry actions included overly aggressive movements (e.g., showing fists, stamping feet, etc.) may have produced a stronger feeling of threat in observers compared to the angry gait pattern. Our results did reveal an interaction between gender and phase in pSTS and Occ across all emotional gait conditions. Both areas are thought to be involved, respectively, in face-body integration [86] and perception of gender information from biological motion [87]. We argue that the gender x phase interaction primarily results from differences in how brain activation unfolds over time between male and female stimuli rather than actual differences in activation amplitude between male vs. female stimuli. For instance, the Occ activity was similar between male and female stimuli in the early and middle LPP but was lower for male vs. female pedestrians in the late LPP phase. Our analysis of this waveform in Fig 5 further showed that while the maximal activation in Occ is similar between male and female stimuli, the signal amplitude drops rapidly in the late LPP phase for male pedestrians, especially in the angry gait. Although not statistically significant, we found a similar pattern of rapid decrease of brain activity in other ROIs as well, especially for the angry male condition. This effect may be caused by the participants disengaging from the task earlier when presented with an angry male pedestrian vs. their female counterpart and other emotional conditions because this specific condition pairing was processed faster. While we cannot ascertain this hypothesis as the experimental design did not allow for reaction time measurement, and while our response accuracies were subtly but significantly higher for the female pedestrian, as reported in the literature [38], shorter reaction times was reported when identifying facial emotions of anger in male vs. female actors [88]. Other studies have also shown that selective attention can modulate the ERR response because of disengagement from a task, causing a decrease in the ERR amplitude [89–91]. Taken together, our results suggest subtle differential effects in the time course of the brain signals in response to emotional gait movements of male vs. female pedestrians rather than actual differences in the maximal amplitude of brain activations.

### Limitations

The patterns of brain activation observed in this study could be influenced by task demands and therefore not fully reflect what one would experience under natural, spontaneous conditions. Analyses were further focussed exclusively on the short-term processing of the emotional gait stimuli. The time window that was examined, however, is consistent with the time required to initiate locomotor trajectory adjustments within the context of pedestrian interactions [92, 93]. The influence of learning effect on either behavioral or neurological results was not considered in the study; however, the increasing accurate responses were not observed between habituation trials and blocks, which again explains the simplicity of the task. The intensity of the emotion was not controlled in this study, and whether the included emotions could be distinguished from other emotions such as fear, disgust, or surprise, was not examined. We did make sure, however, that the emotions were recognized by all participants with a minimum of 80% accuracy and we included only correct trials in our analyses. In addition, the stimuli did not enable the dissociation between the impact of gait patterns (kinematics) and the physical appearance (face or body shape) of the virtual pedestrian for assessing the effect of gender. Further research could replicate this study and employ a neutral gender virtual agent as an additional control condition. Lastly, the inclusion of right-handed participants only, as well as the limited range of emotions studied and the binary classification of sex and gender considered, may limit the generalization of the reported findings.

## Conclusion

We aimed to advance the understanding of spatiotemporal characteristics of brain activity in response to emotional gait using MEG. We used a scenario presentation context with virtual pedestrians, which improved the ecological validity and familiarity of the task. The findings of this study provide evidence that emotional gait, as rendered by stimuli that comprise of biological motion and pictorial information, modulate brain activation within areas associated with biological motion, form, and emotion processing and that the temporal characteristics of the brain signals differ depending on whether the emotions are conveyed by a male or a female individual. Current findings further generate a basis for comparison to identify defective brain processes associated with social cognition deficits in populations with traumatic brain injury, autism spectrum disorder, and schizophrenia [10, 94, 95].

## Supporting information

S1 File (PDF)

S2 File (PDF)

S3 File (PDF)
