# Supplementary material for: Brain mechanisms involved in the perception of emotional gait: A combined magnetoencephalography and virtual reality study
Source: PLoS One. 2024 Mar 29;19(3):e0299103. doi: 10.1371/journal.pone.0299103 (PMC10980214; doi:10.1371/journal.pone.0299103)
Supplement: S2 File — (PDF) [file pone.0299103.s002.pdf]

Request for Permission to Publish Content under CC-BY License

Dear Rights Holder or Representative,

I have submitted a paper for publication in a PLOS journal, and wish to include the content listed below in the paper. I'm hereby requesting your (or your company's or institution's) permission to include the content in my paper. Please note that all PLOS journals are published under a Creative Commons Attribution License (CC BY), which allows for unrestricted use and distribution, even commercial, as long as attribution is given to the creator or rights holder of the content. See https://creativecommons.org/licenses/by/4.0/.

To grant me permission to use the content in my PLOS paper, please fill in the information below and then scan the completed form and send it to me at my email address.

Thank you.

My name:

Yu-Tzu Wu

My email address:

yu-tzu.wu2@mail.mcgill.ca

Description of the content which I'm seeking permission to use (citation and/or title, and pasted screen shot, if applicable):

The Figure 3 in the thesis article, describing the procedure of the trial.

Link to the Content:

https://www.proquest.com/dissertations-theses/brain-mechanisms-involved-perceptio

* * *

On behalf of myself or the rights holder, I hereby grant the permission sought herein.

Signature of Party Granting Permission:

Yu-Tzu Wu Yu-Tzu Wu

Date:

Dec 27th 2023

Printed Name and Title:

Yu-Tzu Wu
